# Looking for Cancer Clues in Publicly Accessible Databases

**DOI:** 10.1002/cfg.378

**Published:** 2004-03

**Authors:** Djamel Medjahed, Peter F. Lemkin, Gary W. Smythers, David J. Munroe

**Affiliations:** 1 Laboratory of Molecular Technology SAIC–Frederick National Cancer Institute at Frederick PO Box B, 915 Tollhouse Avenue, Suite 211 Frederick MD 21701 USA; 2 Science Applications International Corporation Frederick MD USA

## Abstract

What started out as a mere attempt to tentatively identify proteins in experimental cancer-related 2D-PAGE maps developed into VIRTUAL2D, a web-accessible repository for theoretical pI/MW charts for 92 organisms. Using publicly available expression data, we developed a collection of tissue-specific plots based on differential gene expression between normal and diseased states. We use this comparative cancer proteomics knowledge base, known as the tissue molecular anatomy project (TMAP), to uncover threads of cancer markers common to several types of cancer and to relate this information to established biological pathways.


**Comparative and Functional Genomics**
*Comp Funct Genom* 2004; **5**: 196–200.
Published online in Wiley InterScience (www.interscience.wiley.com). **DOI:** 10.1002/cfg.378

**Conference Review**

### Looking for cancer clues in publicly accessible databases†

Djamel Medjahed[1]*, Peter F. Lemkin[1], Gary W. Smythers[1,2] and David J. Munroe[1,2]
[1] *National Cancer Institute at Frederick, PO Box B, Frederick, MD 21702-1201, USA*
[2] *Science Applications International Corporation, Frederick, MD, USA*

**Correspondence to:
Djamel Medjahed, Laboratory of Molecular Technology, SAIC–Frederick, National Cancer Institute at Frederick, 915 Tollhouse Avenue, Suite 211, Frederick, MD 21701, USA.
E-mail: medjahed@ncifcrf.gov*

† *This article is a US Government work and is in the public domain in the USA.*

Received: 10 November 2003
Revised: 12 December 2003
Accepted: 18 December 2003

### Abstract

**What started out as a mere attempt to tentatively identify proteins in experimental cancer-related 2D-PAGE maps developed into VIRTUAL2D, a web-accessible repository for theoretical pI/MW charts for 92 organisms. Using publicly available expression data, we developed a collection of tissue-specific plots based on differential gene expression between normal and diseased states. We use this comparative cancer proteomics knowledge base, known as the tissue molecular anatomy project (TMAP), to uncover threads of cancer markers common to several types of cancer and to relate this information to established biological pathways. Published in 2004 by John Wiley & Sons, Ltd.**

**Keywords:** **cancer pathways; TMAP; VIRTUAL2D**

### Introduction

The recent sequencing and subsequent analysis of the human genome have enabled a paradigm shift: increasingly, efforts are being directed away from a microscopy-based histopathology approach and towards molecular profiling for diagnosis and management of diseases such as cancer. This approach promises to improve the likelihood of positive outcome by early detection and selection of the appropriate therapeutic intervention. One way to achieve this is to use computer-aided pattern recognition algorithms to look for signatures of markers on the basis of significant differential expression between normal and altered states. This formidable challenge relies on high-resolution separation and analysis methods.

Despite being gradually complemented and sometimes replaced by liquid chromatography (LC) techniques, two-dimensional polyacrylamide gel electrophoresis (2D-PAGE) [9] has enjoyed remarkable staying power. In the span of three decades, it has evolved from a multi-process, labour-intensive separation technique to an automated, highly reproducible and sensitive tool that has often been at the core of efforts aimed at the detection of disease-specific biomarkers. However, large-scale identification of proteins remains a pricey proposition, as it is typically carried out in conjunction with tandem mass spectrometry (MS), driving the cost per spot to exceed several hundred Euros. SWISS2D is the largest publicly accessible repository of such data, yet the number of identified spots represents less than a few per cent of the full complement of proteins predicted by the genome sequencing projects. We constructed two databases that address some of these issues, more specifically intended to:

- Facilitate the protein-to-spot assignment in experimental 2D-PAGE maps.
- Optimize experimental conditions by estimating the range of pI and MW attributes in advance.

### Materials and methods

### VIRTUAL2D

VIRTUAL2D [7] is the collection of web-accessible, fully interactive, isoelectric focusing point/molecular mass (pI/MW) charts. These maps have been assembled from primary sequence contained within the combined SWISS-PROT/TrEMBL curated proteome databases [11]. Starting with the datasets in FASTA format released by the European Bioinformatics Institute, electrophoretic and mass attributes are computed for unmodified proteins (save for signal peptides if they are present) using the following approach:

- Scan the primary sequence of the peptide.
- Assign the pK of each contributing amino acid and average over the entire peptide.
- Sum up all the mass contributions.

The resulting pI/Mw is then given by the ratio of:

$$pI_{tot} = \frac{\left(pK_{Cterm} + \sum \text{int } pK_{int} + pK_{Nterm}\right)}{(n+2)} \quad (1)$$

and

$$MW_{tot} = \sum_{i} MW_{i}$$

where the pK and mass values used are the same as in (1).

These attributes; a database Accession No. (GenBank, SWISS-PROT), protein name and cgi requests, are assembled into tab-delimited (ASCII-format) files, which are then processed by a JAVA-based graphical user interface adapted from PtPlot [10].

In the course of building these plots, a bi-modal distribution, centred on either side of a relatively 'depleted' region around pH 7.4, was seen to be conserved for all organisms. Randomly generated sequences varying in length from 50 to 600 amino acids yielded a similar distribution, consistent with a limited pK-segregated proteomic alphabet: roughly half the internal contributing amino acids are acidic, while the other half is basic. Just as important is the fact that none of them have a pK value near the depleted region around pH 7.2.

When launching or accessing VIRTUAL2D, a left panel is presented that contains a list of available organisms, which, when selected, will produce an initial pI/MW map containing all the entries found in the data file. One can zoom in on an area of interest and click on any spot to be transported by hyperlink to a database of choice (default is SWISS-PROT).

To date, the pI/MW charts for 92 organisms have been assembled from data extracted from the published datasets. The central repository can be accessed at **http://ncisgi.ncifcrf.gov/medjahed/** or can be requested from the author and run on a JAVA-enabled web browser.

Comparisons of predicted and experimental charts have yielded mixed results. For very high-resolution gels of relatively simple organisms such as *Escherichia coli*, a subset of proteins for which the theoretical values are close to their measured counterparts can be identified and, in principle, be used as landmarks to align both datasets. The large number of pre- and post-translational modifications characteristic of more complex, multicellular organisms makes it nearly impossible to assign reliably the protein identity of most spots.

### TMAP

As an extension to the two-dimensional information, we have explored using the frequency of detection or abundance of each transcript in cDNA libraries published in the Cancer Anatomy Genome Project (CGAP) [2] database to develop a set of tissue/histology-specific protein expression maps: the Tissue Molecular Anatomy Project (TMAP) [6].

CGAP was launched in 1996 to standardize sample handling and procurement and to track the molecular changes occurring in cells throughout their progression from the normal to the cancerous state. This effort was further enhanced by the development of laser microdissection technology, leading in principle, to purer cell populations.

The correlation between mRNA abundance and protein expression level is known to be complex and non-linear. The aim here *was not* to address this issue but to simply provide a representation that could be used to carry out a comparative analysis between the different histological states.

The starting point of our data-mining was the list of entries in the CGAP library from which we carry a cross-referencing of the Expressed Sequence Tags (EST) in UNIGENE [12] to extract a tab delimited list of gene products, including their frequency

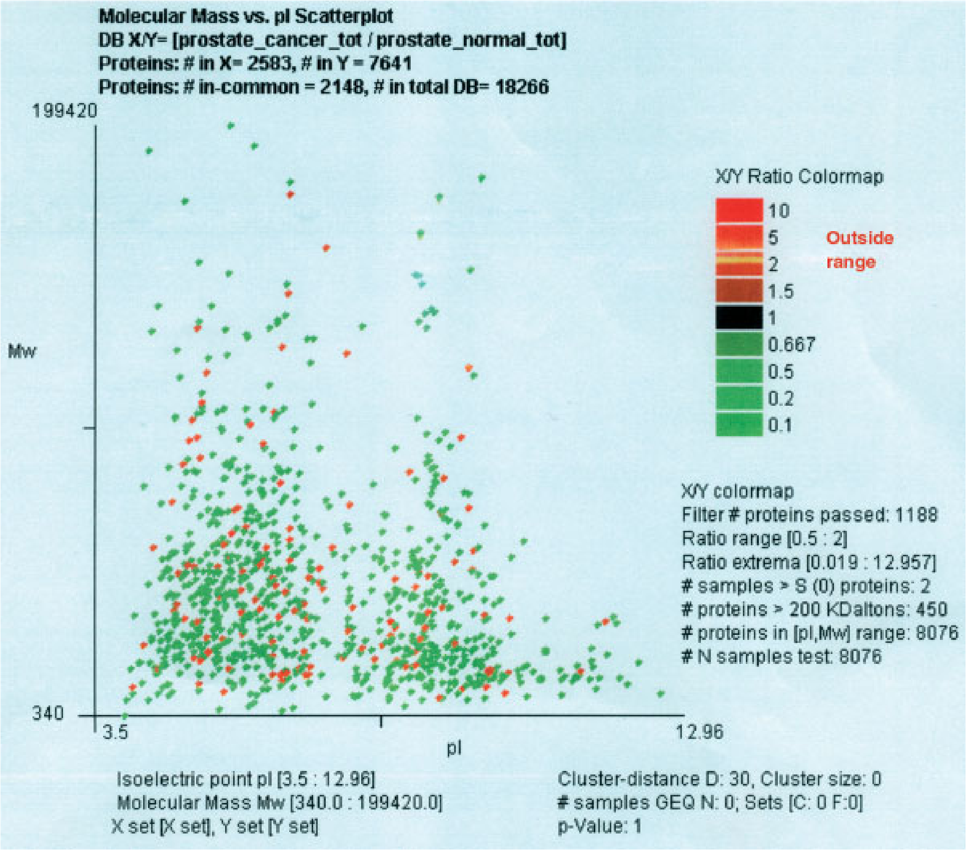

**Figure 1.** A screenshot of TMAP rendering an example pI/MW plot of the differential expression of gene products. The isoelectric focusing point (pI) is along the *x* axis, the molecular mass (MW) is along the *y* axis. 2148 entries are commonly detected in pooled CGAP libraries from prostate cancer and normal (which can also be displayed separately). By dragging the appropriate sliding controls, one can filter the output by selecting a range of differential expression. Here, opting to look only at those entries that are at least two-fold altered reduces the number of displayed proteins to 1188. The expression scale is colour-coded from light green (most downregulated) to bright red (most upregulated). Controls along the axes allow one to zoom in on any part of the chart and upon enabling access to a pre-selected web-based database of choice, one can click on the hyperlinked spots to open a new window containing all the information relevant to the associated protein. Additional filters are accessible under the filter menu and from the graphical user interface. The plot and a tab-delimited report can be output for further examination and/or collaborations of detection within that library. Those transcripts, clustered with genes of known function, have their symbol cross-referenced against the SWISS-PROT database. For small sets of proteins, the pI/MW server [3] can be interrogated to produce isoelectric focusing point and mass values. For large proteome datasets containing tens of thousands of entries, a perl script was developed, which is run locally, to overcome any restrictions and inherent Internet speed bottlenecks. It will extract from the database a flat file with Accession No., primary amino acid sequence and other attributes such as putative function, pathways, etc. It then computes the mass and isoelectric focusing point, as outlined earlier.

Once again, the data file is a simple tab-delimited format with the associated expression information. The frequency of detection of each gene product is used to derive normalized expression levels for each library, so that the most abundant always has a relative expression level of 1.0. The user can select a grey-scale or colour-coded display of this information.

Protplot is the software used to display these expression maps. It has been adapted from MAExplorer, an open-source JAVA-based microarray data analysis suite [8]. It checks and loads all the corresponding files having a .prp extension present within the start directory. Any one of these or a combination thereof can be selected for display.

In some cases, a query of the CGAP database yields more than one library satisfying the search criteria. This is useful in checking the variability of a gene product across similar libraries. One can then pool the results of some or all of the libraries corresponding to the same tissue type and increase the signal : noise ratio. As in any counting experiment, noise increases as $n$ while true signal increases as $n^2$.

Several filters can be applied to these inferred protein expression maps to restrict the number of displayed gene products and monitor their expression profile across several tissues.

A word of caution to potential users: the significance of these comparisons hinges on the quality of the information in the underlying databases, a theme of growing importance in light of the proliferation of biological databases. Although the entire dataset can be displayed, a transcript has to have been detected five or more times ($p < 0.05$) in order to be deemed reliable enough to be included in any comparative analysis. Not all libraries are equally rich in their content and not all the expression data contained within them is of the same quality.

Analysis of differential expression can be carried out between similar libraries, different disease/histological states and indeed different tissues by dividing the normalized expression levels commonly found in both (Figure 1).

cDNA libraries obtained from microdissected prostate samples contain some of the better quality CGAP datasets. Several of them originate from the same patient and span the normal, precancerous and cancerous state. In order to establish a transcript-based model of the progression of the disease, it helps to classify the plausible scenarios. Given three possible histology states, the expression level of any gene product has to adhere to one of the nine following cases in the progression of the disease:

1. Remain constant from normal to pre-cancer to cancer.
2. Remain constant from normal to pre-cancer and increase from pre-cancer to cancer.
3. Remain constant from normal to pre-cancer and decrease from pre-cancer to cancer.
4. Increase from normal to pre-cancer and remain constant from pre-cancer to cancer.
5. Decrease from normal to pre-cancer and remain constant from pre-cancer to cancer.
6. Increase from normal to pre-cancer and decrease from pre-cancer to cancer.
7. Decrease from normal to pre-cancer and increase from pre-cancer to cancer.
8. Increase from normal to pre-cancer and increase from pre-cancer to cancer.
9. Decrease from normal to pre-cancer and decrease from pre-cancer to cancer.

This bookkeeping allows one to go beyond the simple grouping of co-regulated proteins and investigate inverse correlations as well. Relations such as these are numerous and well documented in the literature. In the context of cancer, one such example is p27, a putative tumour-suppressor, which is *downregulated* in most human prostate cancers. In parallel, Skp2, a component of the Skp1–Cul1–F-box protein (SCF) ubiquitin ligase complex, was observed to be *overexpressed* in the same samples leading to the hypothesis, and subsequent experimental confirmation, that degradation of the former is at least partially due to the latter [5].

To date, this comparative cancer proteomics approach has been applied to more than 14 tissues representing the normal, precancer and cancer histological states. This database contains more than 18 000 gene products.

### Discussion

We have presented two proteomic databases: VIRTUAL2D and its extension, TMAP, which attempt to go beyond mere transcript counting by adding functional enhancements to the analysis tools, such as $p$ value filters, library-pooling, etc.

It is critical when interpreting differential gene expression datasets to use statistically sound analysis tools that take into account their reproducibility and validity. In addition to exploring ways to model *de novo* functional biological relationships, we are in the early stages of exploring ways to map the expression data onto established pathways, an example of which is displayed in Figure 2. It is the hope of the present authors that as the amount and quality of information in databases improves, tools such as VIRTUAL2D and TMAP can facilitate the formulation of biological hypotheses.

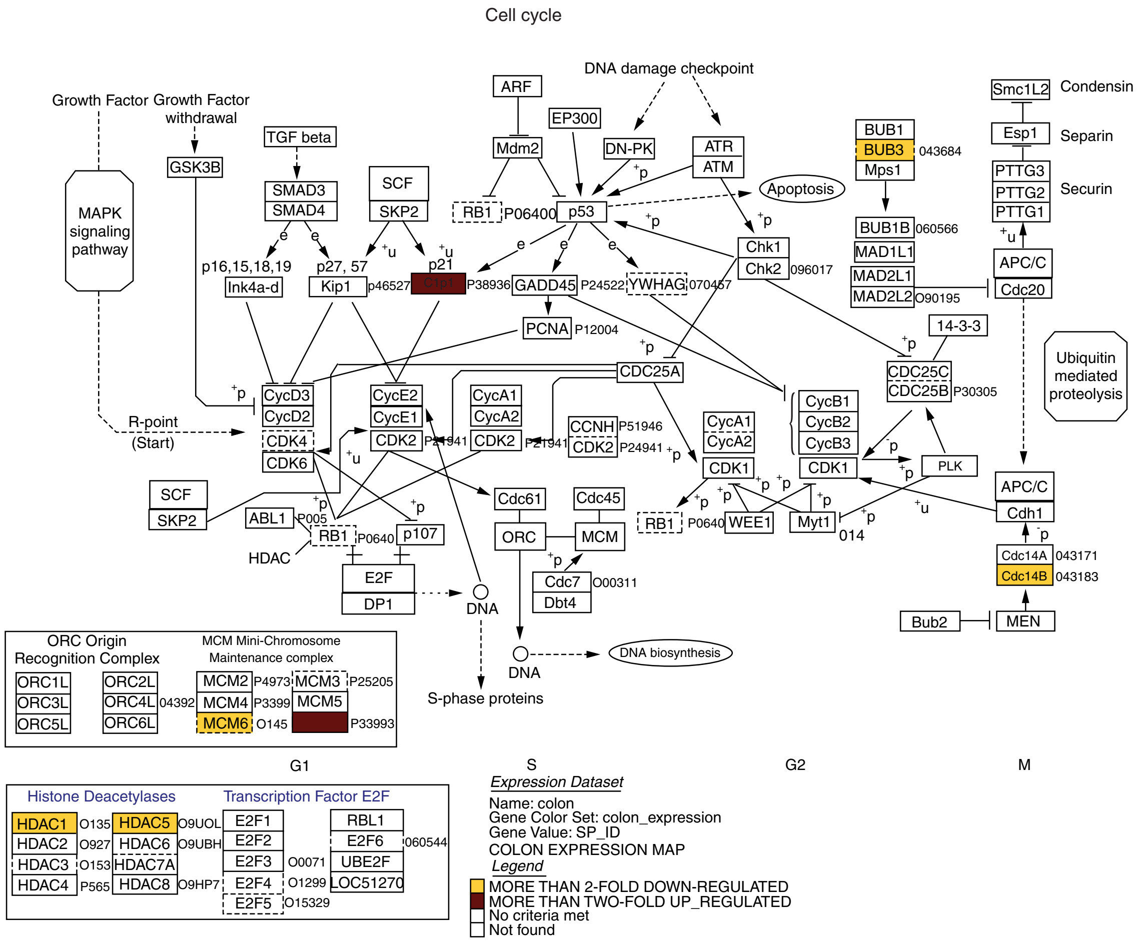

**Figure 2.** For the commonly shared gene products, differential expression can be examined by displaying the colour-coded ratios between the diseased state and the normal state. Using the GenMAPP package [4], Histone deacetylases found in prostate expression data are colour-coded in the pathway adapted from KEGG and describing the cell cycle according to their level of overexpression (HDAC6, yellow) or downregulation (HDAC1, 3, purple) in cancer vs. normal

### Acknowledgements

The content of this publication does not necessarily reflect the views or policies of the Department of Health and Human Services, neither does mention of trade names, commercial products or organization imply endorsement by the US Government. This project has been funded with Federal funds from the National Cancer Institute, National Institutes of Health, under Contract No. NO1-CO-12400.

### References

1. Bjellqvist B, Sanchez JC, Pasquali C, *et al.* 1993. Micropreparative two-dimensional electrophoresis allowing the separation of samples containing milligram amounts of proteins. *Electrophoresis* **14**: 1375–1378.
2. Cancer Genome Anatomy Project: **http://CGAP.nci.nih.gov**.
3. Compute pI/Mw tool: **http://us.expasy.org/tools/pi_tool.html**.
4. GenMAPP: **http://www.genmapp.org**.
5. Lim MS, Adamson A, Lin Z, *et al.* 2002. Expression of Skp2, a p27(Kip1) ubiquitin ligase, in malignant lymphoma: correlation with p27(Kip1) and proliferation index. *Blood* **100**: 2950–2956.
6. Medjahed D, Luke BT, Tontesh TS, *et al.* 2003. Tissue Molecular Anatomy Project (TMAP): an expression database for comparative cancer proteomics. *Proteomics* **3**: 1445–1453.
7. Medjahed D, Smythers GW, Powell AD, *et al.* 2003. VIRTUAL2D: a web-accessible predictive database for proteomics analysis. *Proteomics* **3**: 129–138.
8. MicroArray Explorer: **https://sourceforge.net/projects/maexplorer**.
9. O'Farrell PH. 1975. High resolution two-dimensional electrophoresis of proteins. *J Biol Chem* **250**: 4007–4021.
10. PtPlot: **http://ptolemy.eecs.berkeley.edu/java/ptplot/**.
11. SWISSPROT can be accessed at **http://www.expasy.ch**.
12. UniGene database: **http://www.ncbi.nlm.nih.gov**.

## References

[PDF_00557] Bjellqvist B., Sanchez J. C., Pasquali C., Ravier F., Paquet N., Frutiger S., Hughes G. J., Hochstrasser D. (1993). Micropreparative two-dimensional electrophoresis allowing the separation of samples containing milligram amounts of proteins.. Electrophoresis.

[PDF_00564] Lim Megan S., Adamson Ann, Lin Zhaosheng, Perez-Ordonez Bayardo, Jordan Richard C. K., Tripp Sheryl, Perkins Sherrie L., Elenitoba-Johnson Kojo S. J. (2002). Expression of Skp2, a p27(Kip1) ubiquitin ligase, in malignant lymphoma: correlation with p27(Kip1) and proliferation index.. Blood.

[PDF_00568] Medjahed Djamel, Luke Brian T., Tontesh Tawady S., Smythers Gary W., Munroe David J., Lemkin Peter F. (2003). Tissue Molecular Anatomy Project (TMAP): an expression database for comparative cancer proteomics.. Proteomics.

[PDF_00571] Medjahed Djamel, Smythers Gary W., Powell Douglas A., Stephens Robert M., Lemkin Peter F., Munroe David J. (2003). VIRTUAL2D: A web-accessible predictive database for proteomics analysis.. Proteomics.

[PDF_00576] O'Farrell P. H. (1975). High resolution two-dimensional electrophoresis of proteins.. J Biol Chem.

